# Models for Antitubercular Activity of 5′-*O*-[(*N*-Acyl)sulfamoyl]adenosines

**DOI:** 10.3797/scipharm.1006-03

**Published:** 2010-08-13

**Authors:** Rakesh K. Goyal, Harish Dureja, Gajendra Singh, Anil Kumar Madan

**Affiliations:** 1 Faculty of Pharmaceutical Sciences, Pt. B.D. Sharma University of Health Sciences, Rohtak,124 001, India; 2 Faculty of Pharmaceutical Sciences, M. D. University, Rohtak, 124 001, India

**Keywords:** Wiener’s topochemical index, Molecular connectivity topochemical index, Superpendentic topochemical index, Pendentic eccentricity index, Antitubercular activity, 5′-O-[(N-Acyl)sulfamoyl]adenosines

## Abstract

The relationship between topological indices and antitubercular activity of 5′-*O*-[(*N*-Acyl)sulfamoyl]adenosines has been investigated. A data set consisting of 31 analogues of 5′-*O*-[(*N*-Acyl)sulfamoyl]adenosines was selected for the present study. The values of numerous topostructural and topochemical indices for each of 31 differently substituted analogues of the data set were computed using an in-house computer program. Resulting data was analyzed and suitable models were developed through decision tree, random forest and moving average analysis (MAA). The goodness of the models was assessed by calculating overall accuracy of prediction, sensitivity, specificity and Mathews correlation coefficient. Pendentic eccentricity index – a novel highly discriminating, non-correlating pendenticity based topochemical descriptor – was also conceptualized and successfully utilized for the development of a model for antitubercular activity of 5′-*O*-[(*N*-Acyl)sulfamoyl]adenosines. The proposed index exhibited not only high sensitivity towards both the presence as well as relative position(s) of pendent/heteroatom(s) but also led to significant reduction in degeneracy. Random forest correctly classified the analogues into active and inactive with an accuracy of 67.74%. A decision tree was also employed for determining the importance of molecular descriptors. The decision tree learned the information from the input data with an accuracy of 100% and correctly predicted the cross-validated (10 fold) data with accuracy up to 77.4%. Statistical significance of proposed models was also investigated using intercorrelation analysis. Accuracy of prediction of proposed MAA models ranged from 90.4 to 91.6%.

## Introduction

In the pharmaceutical industry, much effort is being devoted to develop new drugs [1]. The seven steps involved in the drug discovery process are: disease selection, target hypothesis, lead identification, lead optimization, pre-clinical trial, clinical trial and pharmacogenomic optimization. Traditionally, these steps are carried out sequentially, and if one of these steps is slow, it naturally slows down the entire process [2]. Considering both, the potential benefits to human health and the enormous cost in time and money of drug discovery, any tool or technique that enhances the efficiency of any stage of drug discovery enterprise will be highly prized [3]. A viable solution to this quagmire lies in the estimation of necessary properties of molecules directly from their structure without the input of any other experimental data through quantitative structure-activity relationship (QSAR) models [4]. The main hypothesis in the QSAR/QSPR (quantitative structure-activity/property relationship) approach is that all properties (physico-chemical and biological) of a chemical substance are statistically related to its molecular structure [5]. Quantitative relations generated from such studies help in hypothesizing important contributions of specific structural aspects or chemical interactions in modifying physico-chemical properties and biological activities and also in predicting properties and activities of untested and not yet synthesized compounds [6]. Mathematical descriptors of molecular structure, such as various topological indices (TIs), have been widely used in structure-property-activity relationship studies [7]. Topological descriptors are mathematical entities encoding molecular graphs composed of vertices (corresponding to the atoms) and edges (representing the bonds among atoms). These are two-dimensional descriptors which take into account the internal atomic arrangement of compounds, and encode in numerical form information about molecular size, shape, branching, presence of heteroatoms and multiple bonds [8]. One of the most interesting advantages of molecular topology is the straightforward calculation of topological descriptors [9] without requirement of any experimentally derived measurement. The usefulness of TIs in QSPR and QSAR studies has been widely demonstrated, and they have also been used as a measure of structural similarity or diversity by their application to databases virtually generated by computer [10]. Though a large number of topostructural and topochemical indices of diverse nature have been reported in literature but only a small proportion of them has been successfully employed in structure- activity- relationships (SARs). Some of the topostructural and topochemical indices, which have been successfully employed in SAR studies include *Wiener’s index* [11], *Hosoya’s index* [12], *Randic’s molecular connectivity index* [13], *Zagreb group parameters* [14, 15], *Balaban’s index* [16], *Schultz’index* [17], *molecular connectivity topochemical index* [18, 19], *eccentric connectivity index* [20], *revised Wiener index* [21], *E-state index* [22], *eccentric connectivity topochemical index* [23], *Zagreb topochemical indices* [24], and *superaugmented eccentric connectivity indices* [25].

Tuberculosis (TB), one of the oldest recorded human afflictions, is still one of the biggest killers among the infectious diseases, despite the worldwide use of a live attenuated vaccine and combination of several antibiotics [26]. The disease spreads more easily in over crowded places and in the conditions of malnutrition and poverty; characteristics typical of developing countries. Tuberculosis is the commonest opportunistic disease in persons infected with human immunodeficiency virus [27]. *Mycobacterium tuberculosis*, the causative agent of TB, is the leading bacterial cause of infectious disease mortality. *Mycobacterium tuberculosis* and *Yersinia pestis,* the causative agent of plague, have been reported to be pathogens with serious ongoing impact on global public health and potential use as agents of bioterrorism [28]. The development of *M. tuberculosis* strains which are resistant to all of the current front-line antitubercular drugs has prompted worldwide efforts to develop new antibiotics to treat this notorious pathogen [29]. It is well known fact that iron is a required element for growth and survival of *M. tuberculosis* in its host, and iron overload can be an exacerbating cofactor to tuberculosis [30]. Although, iron’s abundance in the earth’s crust, spin state, and redox tuneability makes it the most versatile among transition elements, the insolubility of ferric hydroxide at pH 7.4 limits the concentration of [Fe^3+^] (the free aqueous ion) to ∼10^−18^ M. However, even below this concentration, free ferric ion is toxic. To avoid toxicity and regulate iron transport, the human serum iron transport protein, transferrin, maintains the free ferric iron concentration at about 10^−24^ M [31]. In a mammalian host, the concentration of free iron in serum and body fluids is too low to support growth of bacteria [32]. The ability of pathogens to obtain iron from transferrins, ferritin, hemoglobin, and other iron-containing proteins of their host is central to whether they can live or die [33]. Both pathogenic and saprophytic microorganisms have evolved sophisticated iron-acquisition systems to overcome iron deficiency imposed by host defensive mechanism and their environment. At the core of such systems is the production of small molecules known as siderophores, which are secreted into the extracellular space, tightly bind available iron, and then are reinternalized with their bound iron through specific cell surface receptors [34]. *M. tuberculosis* is reported to produce two series of structurally related siderophores, collectively known as the mycobactins, which are critical for virulence and growth. Mycobactin biosynthesis is initiated by MbtA, an adenylate-forming enzyme that catalyzes a two-step reaction and is responsible for incorporating salicylic acid into the mycobactins [35]. The reaction mechanism catalyzed by MbtA provides several opportunities to develop inhibitors against MbtA [32]. MbtA is an ideal target since it has no mammalian homologues [36]. Inhibition of siderophore biosynthesis has emerged as an attractive strategy to develop new antibiotics against pathogens which require siderophores for virulence [32].

In the present study, a pendenticity based topochemical descriptor termed as *pendentic eccentricity index* (in both topostructural and topochemical forms) has been conceptualized and successfully utilized along with existing TIs for development of models for prediction of antitubercular activity of 5′-*O*-[(*N*-Acyl)sulfamoyl]adenosines.

## Methodology

### Dataset

**Fig. 1.**
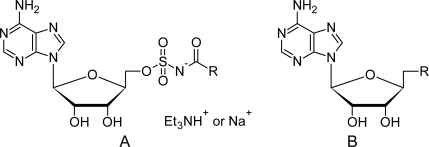
Basic structures of 5′-*O*-[(*N*-Acyl)sulfamoyl]adenosines [35].

A dataset comprising of 31 analogues of 5′-*O*-[(*N*-Acyl)sulfamoyl]adenosines was selected for the present investigation [35]. The basic structures of 5′-*O*-[(*N*-Acyl)sulfamoyl]-adenosines are shown in Fig. 1 and the various substituents have been enlisted in Tab. 1. Somu *et al.* reported that, in order to enhance stability, all compounds were converted to triethylammonium salts after purification while conversion to alkali salts was readily achieved through ion-exchange [36]. In present study, only basic structures were taken into consideration while determining index values.

### Enzyme Assay and Biological Activity against Whole-cell M. tuberculosis

Enzyme assays were performed by Qiao *et al.* [35] at 37 °C with recombinant MbtA expressed in *E. coli* in a buffer of 75 mM Tris-HCl, PH 7.5, 10mM MgCl_2_, 2 mM DTT, 250 μM salicylic acid, 10 mM ATP, and 1 mM PPi. The apparent inhibition constants (K*i*^app^) were determined by fitting the concentration-responce plots either to the Hill equation or to the Morrison equation. All of the K*i*^app^ values reported therein are uncorrected for substrate concentrations and represent an upper limit of the true dissociation constant. Although, the K*i*^app^ reorted are not a measure of the true inhibitor potency, the differences are reflective of free energy differences associated with inhibitor binding to Mbta, presuming equivalent modalities of inhibition [35]. All inhibitors were also evaluated against whole-cell *M. tuberculosis* H37Rv under iron-limiting and iron-rich conditions by Qiao *et al.* [35].

For the purpose of present study, the analogues possessing K*i*^app^ values of ≤0.05 μM were considered to be active and analogues possessing K*i*^app^ values of >0.05 μM were considered to be inactive. Further, the analogues possessing MIC_99_ (Minimum inhibitory concentration that inhibited >99% of cell growth) values of ≤12.5 μM in iron-deficient conditions and ≤50 μM in iron-rich conditions were considered to be active, and analogues possessing MIC_99_ values of >12.5 μM in iron-deficient conditions and >50 μM in iron-rich conditions were considered to be inactive.

### Topological indices

Values of twenty-six topological indices [13–15, 18–20, 23–25, 37–50] of diverse nature used in the present study (Tab. 2) were calculated for all the analogues involved in the data set using an in-house computer program.

### Decision tree

The decision tree (DT) methodology determines activity of a chemical through a series of rules based on selection of descriptors [51]. The simplified mechanism of a decision tree is to find some rules for each class based on the descriptors of the training set. These rules are subsequently utilized for building a decision tree having several branches leading to a leaf with a given class assignment [52]. The name decision tree is due to the reason that the classification is done using a set of tests (or decisions) that are arranged in the form of a tree [53]. The prediction for a molecule reaching a given terminal node is obtained by majority vote of the molecules reaching the same terminal node in the training set. The tree with lowest value of error in cross-validation is selected as optimal tree [54]. In this study, R program (version 2.1.0) along with RPART library was used to grow decision tree.

### Random Forest

A random forest (RF) is an ensemble of unpruned classification trees created by using bootstrap samples of the training data to construct multiple trees (forests) and random subsets of variables to define the best split at each node, hence the name “random” forests [55, 56]. Random forest operates by generating a user-defined number of decision trees, 100 in this application. Mathematically a RF may be expressed as [57]

$$\text{R}=\{{\text{T}}_{1}(\text{X}),\;{\text{T}}_{2}(\text{X})\;-------\;{\text{T}}_{B}(\text{X})\}$$

Where T_1_(X) is a single decision tree and X represents a single molecular descriptor vector. In present study, the RFs were grown with the R program (version 2.1.0) using the random forest library.

### Moving average analysis

In order to develop single topological index based models for classifying data set into active and inactive analogues, moving average analysis (MAA) was applied. Index values of all the 26 chosen descriptors were analyzed and suitable models were developed after identification of the active ranges by maximization of moving average with respect to active compounds (<35% = inactive, 35–65% = transitional, >65% = active) [44, 54]. Subsequently, each analogue of data set was assigned a biological activity using these models, which was then compared with the reported activity [35]. The apparent inhibition constant was reported quantitatively as K*i*^app^ (μM) at different concentrations. The analogues possessing K*i*^app^ values of ≤0.05 μM were considered to be active [labelled as “A” (N=10)] and analogues possessing K*i*^app^ values of >0.05 μM were considered to be inactive [labelled as “B” (N=21)] for the purpose of present study. The analogues possessing MIC_99_ (Minimum inhibitory concentration that inhibited >99% of cell growth) values of ≤12.5 μM in iron-deficient conditions and ≤50 μM in iron-rich conditions were considered to be active, and analogues possessing MIC_99_ values of >12.5 μM in iron-deficient conditions and >50 μM in iron-rich conditions were considered to be inactive for the purpose of present study.

### Calculation of topological indices

Though a total of 26 indices were employed for the present study (Tab. 2) but 11 indices were ultimately shortlisted by either DT or MAA. Classification ability and non-correlation nature of TIs were the main criteria adopted for short listing of TIs for MAA.

### Wiener’s topochemical index(W_c_)

*Wiener’s topochemical index* [41] is defined as the sum of the chemical distances between all pairs of vertices in hydrogen-suppressed molecular graph. It is a refined form of oldest and widely used distance-based topological index – Wiener’s index [11] and this modified index takes into consideration the presence as well as relative position of heteroatom(s) in a molecular structure. It can be expressed as:

Eq. 1. $$W_{c}=\frac{1}{2}\sum_{i=1}^{n}\sum_{j=1}^{n}P_{i_{c}j_{c}}$$

where *P_i_c_j_c__* is the chemical length of the path that contains the least number of edges between vertex *i* and *j* in the graph *G*, *n* is the number of vertices in the hydrogen depleted graph [41].

### Molecular connectivity topochemical index (χ^A^)

The *molecular connectivity topochemical index* [18, 19] is defined as the summation of the modified bond values of adjacent vertices for all edges in the hydrogen-suppressed molecular graph. It is a modified form of the widely used adjacency-based topological index – *molecular connectivity index* [13, 43] and it takes into consideration the presence as well as relative position of heteroatom(s) in a molecular structure, as per the following equation:

Eq. 2. $$\chi^{A}=\sum_{i=1}^{n}{\left(V_{i}^{c}V_{j}^{c}\right)}^{-\frac{1}{2}}$$

where n is the number of vertices, $V_{i}^{c}$ and $V_{j}^{c}$ are the chemical degrees of adjacent vertices i and j forming the edge {i, j} in a graph *G*. The modified degree of a vertex can be obtained from the adjacency matrix by substituting row element corresponding to heteroatom, with relative atomic weight with respect to carbon atom [18, 19].

### Superpendentic index (∫^P^)

A pendenticity based graph invariant termed as *superpendentic index* and denoted by ∫*^P^* is calculated as the square root of the sum of products of the non-zero row elements in the pendent matrix [49]. It is expressed as:

Eq. 3. $$\int^{P}={\left[\sum_{i=1}^{n}\prod_{j=1}^{m}P(ij)\right]}^{0.5}$$

Similarly, its topochemical version termed as *superpendentic topochemical index* ( $\int_{c}^{P}$) can be calculated from chemical pendent matrix as:

Eq. 4. $${\int_{c}^{P}=\left[\sum_{i=1}^{n}\prod_{j=1}^{m}P_{c}(ij)\right]}^{0.5}$$

where *m* and *n* are maximum possible numbers of *i* and *j* respectively.

### Pendentic eccentricity index (ξ^P^)

*Pendentic eccentricity index* (ξ*^P^*), proposed in the present study, can be defined as the summation of the quotients of the product of non-zero row elements in the pendent matrix and squared eccentricity of the concerned vertex, for all vertices in the hydrogen suppressed molecular graph. Pendent matrix, *Dp*, of a graph *G* is a submatrix of distance matrix obtained by retaining the columns corresponding to pendent vertices i.e. terminal vertices or an end vertex with a degree of one [58]. The eccentricity *E_i_* of a vertex *i* in a graph *G* is the path length from vertex *i* to the vertex *j* that is farthest from *i* (*Ei* = max d(*ij*); j *G*) It is expressed as:

Eq. 5. $$\xi^{P}=\sum_{i=1}^{n}\left\{\prod_{j=1}^{m}P_{\left(ij\right)}/E_{i}^{2}\right\}$$

where *P*_(*ij*)_ is the length of the path that contains the least number of edges between vertex *i* and vertex *j* in graph *G; n* is the number of vertices in the hydrogen depleted graph.

Similarly topochemical version of ξ*^P^* - *pendentic eccentricity topochemical index* ( $\xi_{c}^{P}$) can be expressed as:

Eq. 6. $$\xi_{c}^{P}=\sum_{i=1}^{n}\left\{\prod_{j=1}^{m}P_{\left(i_{c}j_{c}\right)}/E_{i_{c}}^{2}\right\}$$

where *P*_(*i_c_j_c_*)_ is the chemical length of the path that contains the least number of edges between vertex *i*_c_ and vertex *j_c_* in graph *G; n* is the number of vertices in the hydrogen depleted graph.

**Fig. 2.**
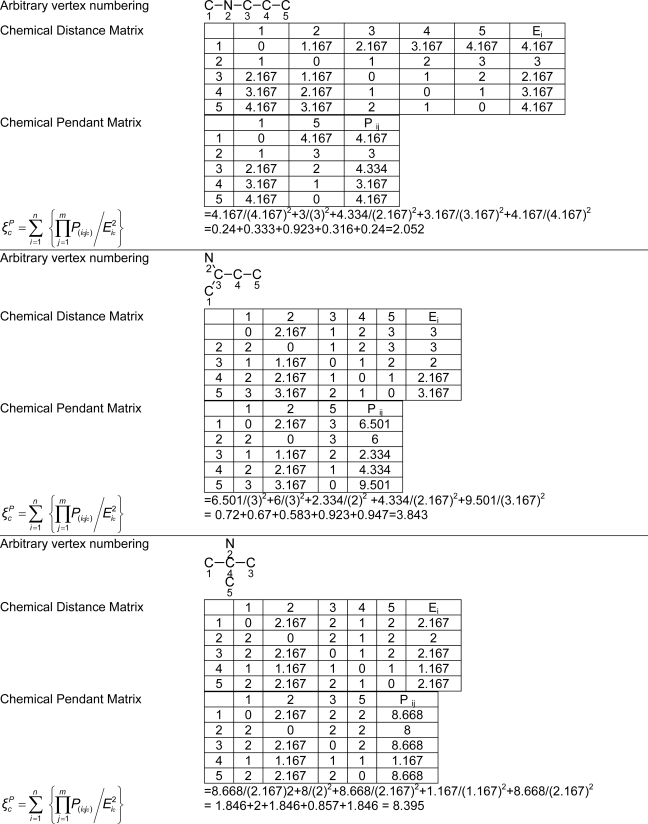
Calculation of *pendentic eccentricity topochemical index* values for three isomers of a five membered molecule containing one heteroatom and at least one pendent vertex.

*Pendentic eccentricity topochemical index* can be easily calculated from chemical pendent matrix*,* a submatrix of chemical distance matrix. Calculation of proposed index for three isomers of five membered molecule containing one heteroatom and at least one pendant vertex is exemplified in Fig. 2. The sensitivity of the proposed topochemical descriptor towards presence and relative position of heteroatom(s) for all three, four and five membered isomers containing only one heteroatom and at least one pendent vertex has been illustrated in Tab. 3. Discriminating power and degeneracy of the *pendentic eccentricity topochemical index* were investigated using all possible structures with three, four and five vertices containing one heteroatom and at least one pendent vertex and were compared with that of the other three indices (Tab. 4).

### Zagreb indices (M_1_and M_2_)

This pair of indices [14, 15] was introduced in 1972 and have been given different names in the literature, such as the Zagreb Group indices, the Zagreb group parameters and most often, the Zagreb indices. These indices are denoted by *M_1_* and *M_2_* and are defined as per the Eqs. 7 and 8:

Eq. 7. $$M_{1}=\sum_{vertices}d(i)d(i)$$

Eq. 8. $$M_{2}=\sum_{edges}d(i)d(j)$$

where *d(i)* is the degrees of vertex i, which can be defined as number of edges incident on a vertex i [58] and *d(i)d(j)* is the weight of edge *{i,j}*.

Similarly *Zagreb topochemical indices* [24] $M_{1}^{c}$ and $M_{2}^{c}$ are defined as per the Eqs. 9 and 10:

Eq. 9. $$M_{1}^{c}(G)=\sum_{i=1}^{n}{\left(d^{c}(i)\right)}^{2}$$

where *d^c^(i)* is chemical degree vertex *i* and *n* is the number of vertices.

Eq. 10. $$M_{2}^{c}(G)=\sum_{ij}^{n}\left(d^{c}(i)d^{c}(j)\right)$$

where *d^c^(i)d^c^(j)* is the chemical weight of the edge *{i,j}* in the hydrogen suppressed molecular graph and *n* is the number of edges [24].

### Augmented eccentric connectivity index (^A^*ξ^c^*)

This is an adjacency-cum-distance based index [44] and is defined as the summation of the quotients of the product of adjacent vertex degrees and eccentricity of the concerned vertex, for all vertices in the hydrogen suppressed molecular graph. It is expressed as:

Eq. 11. $${}^{A}\xi^{c}=\sum_{i=1}^{n}\left(\frac{M_{i}}{E_{i}}\right)$$

where, *M_i_* is the product of degrees of all vertices (*v_j_*), adjacent to vertex *i*, E*_i_* is the eccentricity, and *n* is the number of vertices in graph *G* [44].

### Performance evaluation

The goodness of the models was assessed by calculating sensitivity, specificity [59, 60], overall accuracy of prediction [44], and Matthews correlation coefficient (MCC) [61]. The sensitivity and specificity are defined as per the following:

Sensitivity=TP/(TP+FN),                  Specificity=TN/(TN+FP)

Where the true positive (TP) is the number of compounds correctly predicted as active, false negative (FN) is the number of compounds incorrectly predicted as inactive, true negative (TN) is the number of compounds correctly predicted as inactive, false positive (FP) is the number of compounds incorrectly predicted as active. Thus, the overall accuracy is defined as:

$$\text{Overall}\;\text{accuracy}=\frac{\text{TP}+\text{TN}}{\text{TP}+\text{FN}+\text{TN}+\text{FP}}*100$$

MCC quantifies the strength of the linear relation between the molecular descriptors and the classifications, and it may often provide a much more balanced evaluation of the prediction than, for instance, the percentages (accuracy). Matthews correlation coefficient of 1 corresponds to a perfect prediction, whereas 0 corresponds to a completely random prediction and takes both sensitivity and specificity into account. It is calculated as [59]:

$$MCC=\frac{TP*TN-FN*FP}{\sqrt{\left(TP+FN)*(TP+FP)*(TN+FN)*(TN+FP\right)}}$$

The percent degree of prediction for each range as well as overall degree of prediction were calculated. The percent classification was obtained from the ratio of number of compounds present in active and inactive ranges to the total number of compounds in the data set. The percent degree of prediction for each range as well as overall accuracy of prediction of the proposed model for antitubercular activity in iron-deficient and iron-rich state were also measured.

The validation of the DT based model and self- consistency test were performed by 10-fold cross validation (CV) method, in which the compound dataset was randomly split into 10 folds. The model was developed using 9 randomly selected folds, and prediction was done on the remaining fold. The goodness of DT based model was also assessed by calculating sensitivity, specificity, overall accuracy of prediction and MCC. The 10-fold CV results are given in Tab. 5. From a practical application point of view, topological descriptors used should be least correlated [62]. Absence of direct correlation indicates that the two indices are distinctive and consider different structural components. Statistical significance of TIs used in building predictive models was also assessed by intercorrelation analysis by using index values of analogues of 5′-*O*-[(*N*-Acyl)sulfamoyl]adenosines.

## Results and Discussion

Computational approaches applied in drug discovery and toxicity prediction often require molecular descriptors that reflect structural information and physicochemical properties of molecules [63]. The description of the molecular structure through the so-called molecular descriptors is a more difficult but necessary task. Difficulties arise in the generation of such indices, due to non-mathematical nature of the molecular structure [64]. Topological indices are one of the widely used molecular descriptors, which are easily available and can be quickly computed for existing and virtual structures [65, 66]. The successful implementation of QSPR and QSAR certainly decreases the number of compounds synthesized, by making it possible to select the most promising compounds. However, it does not completely eliminate the trial and error factor involved in the development of new drugs [67].

Researchers are striving hard to develop new TIs with not only high discriminating power but also devoid of both degeneracy and correlation with existing TIs. As observed from Fig. 2, value of *pendentic eccentricity index* changes by >4 times (from 2.052 to 8.395) with a small change in the branching of a five membered molecule containing one heteroatom and at least one pendant vertex. Thus, novel descriptor has high discriminating power, defined as the ratio of highest to lowest value for all possible structures of same number of vertices. This is evident from the fact that the ratio of the highest to lowest value for all possible structures containing five vertices is 6.25 for $\xi_{c}^{P}$, in contrast to 1.5, 1.22 and 2.24 for *W_c_*, χ^A^ and $\int_{c}^{P}$ respectively. Thus, *pendentic eccentricity topochemical index* revealed ∼4 times higher discriminating power with respect to *Wiener’s topochemical index*, >5 times higher discriminating power with respect to *molecular connectivity topochemical index* and ∼2.8 times higher discriminating power with respect to s*uperpendentic topochemical index* for all the possible structures of five vertices containing a heteroatom and at least one pendent vertex (Tab. 4). High discriminating power and extremely low degeneracy are desirable properties of an ideal topological index. High discriminating power of the proposed new descriptor makes it more sensitive towards any change in molecular structure.

Degeneracy is the measure of ability of an index to differentiate between the relative positions of atom in a molecule. It is well known fact that topological indices show degeneracy, that is, two or more non-isomorphic graphs may have identical numerical values for an index [68]. The novel *pendentic eccentricity topochemical index* had significantly reduced degeneracy as compared to *Wiener’s topochemical index* and *superpendentic topochemical index.* This is evident from the fact that *pendentic eccentricity topochemical index* had only 5 identical values out of 30 structures with only five vertices containing one heteroatom and at least one pendent vertex whereas *Wiener’s topochemical index* and *superpendentic topochemical index* had 13 and 9 identical values, respectively, for the same compounds (Tab. 4). It is pertinent to mention here that *pendentic eccentricity topochemical index* had also reduced degeneracy as compared to *molecular connectivity topochemical index,* as is evident from the fact that novel index had a single identical index value out of 31 values of dataset under study, whereas *molecular connectivity topochemical index* had two identical values for the same (see tab. 1). Lower the degeneracy, better is the index [39]. Significant reduction in degeneracy indicates the enhanced capability of novel topochemical index to differentiate and demonstrate slight variations in the molecular structure. This means that the likeliness of different structures to have same value is very less. As observed from Tab. 6, *pendentic eccentricity topochemical index* is not correlated with most of the commonly used TIs. Pairs of indices with r≥0.97 are considerably highly intercorrelated, those with 0.90≥r<0.97 are appreciably correlated, those with 0.50≤r≤0.89 are weakly correlated and finally the pairs of indices with low r values (<0.50) are not intercorrelated [69]. Intercorrelation analysis (Tab. 6) revealed that the pair of indices $\int_{c}^{AP}-\xi_{c}^{P}$ are highly intercorrelated, pair of indices $W_{c}-M_{1}^{c}$, $M_{1}^{c}-M_{2}^{c}$ are appreciably intercorrelated, pair of indices *W_c_* - *χ^A^*, $W_{c}-\int_{c}^{\text{AP}}$, $W_{c}-\xi_{c}^{P}$, $W_{c}-M_{2}^{c}$, $\chi^{A}-M_{1}^{c}$, $\chi^{A}-M_{2}^{c}$, $\int_{c}^{AP}-M_{1}^{c}$, $M_{1}^{c}{-}^{A}\xi^{c}$, $M_{2}^{c}{-}^{A}\xi^{c}$, $\xi_{c}^{P}-M_{1}^{c}$ are weakly correlated and pair of indices *W_c_* - *^A^ξ*^c^, $\chi^{A}-\int_{c}^{AP}$, $\chi^{A}-\xi_{c}^{P}$, χ^A^ - *^A^ξ^c^*, $\int_{c}^{\text{AP}}-M_{2}^{c}$, $\int_{c}^{\text{AP}}-{}^{A}\xi^{c}$, $\xi_{c}^{P}-M_{2}^{c}$, and $\xi_{c}^{P}-{}^{A}\xi^{c}$ are not intercorrelated.

**Fig. 3.**
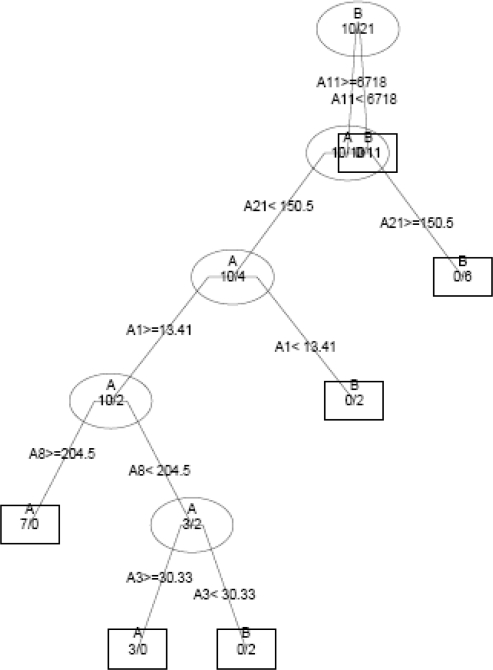
Topology of a decision tree distinguishing active compounds {A} from inactive compounds {B}.

In the present study, DT, RF and MAA based models were developed for the prediction of antitubercular activity of 5′-*O*-[(*N*-Acyl)sulfamoyl]adenosines. The decision tree was built by utilizing 26 TIs of diverse nature. This recursive partitioning scheme generates rules based on the numerical data of the available descriptors for each molecule. In this case, a classification of data set [35] into active and inactive compounds was desired. Decision tree assigns a probability value (0–1) that a compound is active or inactive; compounds with the probability equal to or greater than 0.5 are designated as active, while others are designated as inactive [70]. Decision tree identified five important topological indices: *superpendentic topochemical index* (A11), *Zagreb group parameter, M_2_* (A21), *Molecular connectivity topochemical index* (A1), *Zagreb topochemical index*, $M_{2}^{c}$ (A8) and a*ugmented eccentric connectivity topochemical index* (A3). The obtained topology of the decision tree is shown in Fig. 3, where the respective descriptor is denoted with an alphanumerical abbreviation that refers to Tab. 2. The index at the root node is most important and significance of index decreases as the tree increases. The DT classified analogues of 5′-O-[(N-Acyl)sulfamoyl]adenosines in the training set with an accuracy of 100% and the cross validated set with an accuracy of 77.4% with regard to antitubercular activity. The sensitivity and specificity of DT based model in the training set was found to be 100%. The sensitivity and specificity of decision tree based model in the cross-validated set was of the order of 70% and 80.9% respectively. The values of MCC for DT based model in the training set and cross validated set are 1 and 0.497 respectively suggesting satisfactory performance as well as robustness of the model. The values of sensitivity, specificity and MCC are shown in Tab. 5.

The random forests were also grown utilizing 26 TIs enlisted in Tab. 2. The RF classified 5′-*O*-[(*N*-Acyl)sulfamoyl]adenosines with regard to antitubercular activity with an accuracy of 67.74% and out-of-bag (OOB) estimate of error was 32.26%. The sensitivity, specificity and MCC value of RF based model was found to be 50%, 76.19% and 0.26 respectively. The values of sensitivity, specificity and MCC are shown in Tab. 5.

Using a single index at a time, MAA provided four independent models based on *W_c_*, χ^A^, $\int_{c}^{P}$ and $\xi_{c}^{P}$ with an accuracy of prediction ranging from 90.4% to 91.6%. The index values of various analogues along with their substituents are presented in Table 1. The reason behind choosing these four indices for development of models was that these indices provide structural information on different concepts. *Wiener’s topochemical index* is based upon inter-atomic distances and any increase in linearity and molecular size results in increase in the value of *Wiener’s topochemical index.* M*olecular connectivity topochemical index*, on the other hand, is based upon adjacency or connectivity of atoms within a molecule. S*uperpendentic topochemical index* and novel *pendentic eccentricity topochemical index* are pendenticity based topological indices and thus take into consideration pendent vertices in the molecule

The methodology used in the present study aims at the development of suitable models for providing lead molecules through exploitation of the active ranges in the proposed models based on topological indices. Proposed models are unique and differ widely from conventional QSAR models. Both systems of modeling have their own advantages and limitations. In the instant case, the modeling system adopted has distinct advantage of identification of narrow active range(s), which may be erroneously skipped during routine regression analysis in conventional QSAR modeling. Since the ultimate goal of modeling is to provide lead structures, therefore, these active ranges can play vital role in lead identification [71].

Retrofit analysis of the data with regard to *Wiener’s topochemical index* (Tab. 7–9) revealed that 90.4% analogues were predicted correctly with respect to antitubercular activity. *Extremely low* average K*i value of 0.019 μM* of correctly predicted compounds *indicates high potency of the active range in the proposed model.* Activity of all the analogues in both inactive ranges was predicted correctly. The average K*i*^app^ values for lower inactive and upper inactive ranges were found to be 43.22 μM and 37.05 μM respectively. Existence of a transitional range indicates gradual change in biological activity. The ratio of average K*i*^app^ values of active range with lower inactive range and upper inactive range was found to be 1:2274.73 and 1:1950 respectively for correctly predicted analogues. Overall accuracy of this model, for prediction of antitubercular activity in iron-deficient and iron-rich state was found to be 80.9%. Sensitivity, specificity, and MCC for this model was found to be 100%, 86.66%, and 0.8 respectively.

Retrofit analysis of the data with regard to *molecular connectivity topochemical index* (Tab. 7–9) revealed that 91.3% analogues were predicted correctly with respect to antitubercular activity. *Extremely low average* K*i*^app^ *value of 0.016 μM* of correctly predicted compounds *indicates high potency of the active range in the proposed model.* Biological activity of all the analogues in both inactive ranges was predicted correctly. The average K*i*^app^ values of lower inactive range and upper inactive range were found to be 30.71 μM and 41.5 μM respectively. Existence of a transitional range indicates gradual change in biological activity. The ratio of average K*i*^app^ values of active range with lower inactive range and upper inactive range was found to be 1:1919.37 and 1: 2593.75 respectively for correctly predicted analogues. Overall accuracy of this model, for prediction of antitubercular activity in iron-deficient and iron-rich state was found to be 78%. Sensitivity, specificity, and MCC for this model was found to be 100%, 88.23%, and 0.8 respectively.

Retrofit analysis of the data with regard to *superpendentic topochemical index* (Tab. 7–9) revealed that 91.6% analogues were predicted correctly with respect to antitubercular activity. *Extremely low* average K*i*^app^ *value of 0.018 μM* of correctly predicted compounds *indicates high potency of the active range in the proposed model.* Activity of all the analogues in both inactive ranges were predicted correctly. The average K*i*^app^ values of lower inactive and upper inactive ranges were found to be 44.47 μM and 52.93 μM respectively. Existence of a transitional range indicates gradual change in biological activity. The ratio of average K*i*^app^ values of active range with lower inactive range and upper inactive range was found to be 1:2470.55 and 1:2940.55 respectively for correctly predicted analogues. Overall accuracy of this model, for prediction of antitubercular activity in iron-deficient and iron-rich state was found to be 91.6%. Sensitivity, specificity, and MCC for this model was found to be 100%, 88.23%, and 0.82 respectively.

Retrofit analysis of the data with regard to *pendentic eccentricity topochemical index* (Tab. 7–9) revealed that 91.3% analogues were predicted correctly with respect to antitubercular activity. *Extremely low* average K*i*^app^ *value of 0.018 μM* for the correctly predicted compounds *indicates high potency of the active range in the proposed model.* Activity of all the analogues in both inactive ranges was predicted correctly. The average K*i*^app^ value of lower inactive range and of upper inactive range was found to be 48.9 μM and 52.92 μM respectively. Existence of a transitional range indicates gradual change in biological activity. The ratio of average K*i*^app^ values of active range with lower inactive range and upper inactive range was found to be 1:2716.66 and 1:2940 respectively. Overall accuracy of this model, for prediction of antitubercular activity in iron-deficient and iron-rich state was found to be 82.6%. Sensitivity, Specificity, and MCC for this model has been found to be 100%, 87.5%, and 0.82 respectively.

*Pendentic eccentricity topochemical index* ( $\xi_{c}^{P}$) depends upon number of pendent atoms and eccentricity. It also takes care of both the nature as well as relative position(s) of pendent atom(s)/heteroatom(s). For a compound to be biologically active, two pendent vertices on the cyclic substituent R (at appropriate places) are essential as observed from relative K*i*^app^ (μM) values [35]. Any deviation from such substitution leads to either loss or reduction in biological activity. All of the compounds which have been characterized as active by the proposed model contained two pendent atoms in the cyclic substituent R. Accordingly, all the compounds [excepting 7 and 16] predicted as active by the proposed model were also experimentally reported to be active. Compounds 7 and 16 were categorised as active according to our proposed model with a cut off value of ≤0.05 μM. Though these two compounds were experimentally reported to be inactive as per the proposed model with a cut off value of ≤0.05 μM but both these compounds exhibited significant biological activity with K*i*^app^ values of 0.061 and 0.137 respectively when compared to average K*i*^app^ values of ∼50 μM for the inactive range. Consequently, all the compounds which were categorised as active as per the proposed model were either experimentally reported to be active or exhibited significant biological activity. All the compounds which have been characterized as inactive as per model possessed either less than two pendent atoms or more than two pendent atoms in the cyclic substituent R with an exception of compound 17. Inactivity of compound 17 may be due to lack of pendent vertex at ortho-position. This fact has already been reported earlier [35]. Since study signifies the influence of both the number as well as relative position(s) of pendent atom(s) in the cyclic substituent R on the biological activity, therefore, pendenticity based topological descriptors will naturally be of utmost importance in drug design.

**Fig. 4.**
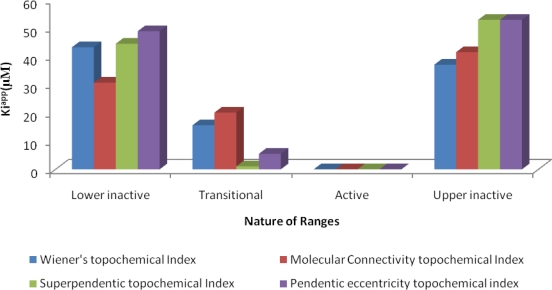
Average K*i*^app^ (μM) values of correctly predicted analogues in various ranges of the proposed MAA topological models.

**Fig. 5.**
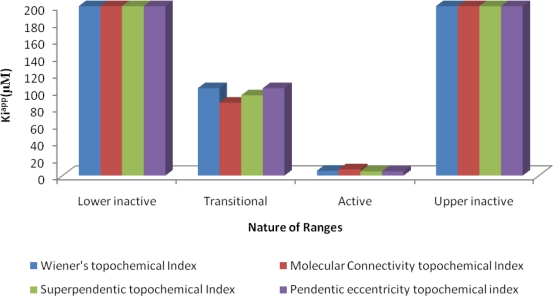
Average K*i*^app^ (μM) values of correctly predicted analogues in various ranges of the proposed MAA topological models in iron-deficient state.

**Fig. 6.**
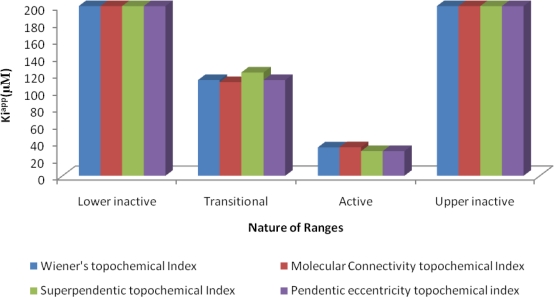
Average K*i*^app^ (μM) values of correctly predicted analogues in various ranges of the proposed MAA topological models in iron-rich state

The results of average K*i*^app^ (μM) values of correctly predicted analogues in various ranges of the proposed MAA based topological models are shown in Figures 4–6.

## Conclusion

*Pendentic eccentricity topochemical index -* a novel molecular descriptor exhibited high discriminating power, sensitivity towards both the presence as well as relative position(s) of pendent/heteroatom(s) apart from reduced degeneracy. Moreover, *Pendentic eccentricity topochemical index* was found not to be correlated with important topological descriptors rendering it highly beneficial tool for isomer discrimination, similarity/dissimilarity, drug design, quantitative structure-activity/structure-property relationships, lead optimization and combinatorial library design.

Significant correlation of topological descriptors with antitubercular activity of 5′-*O*-[(*N*-Acyl)sulfamoyl]adenosines led to development of numerous models through decision tree, random forest and MAA. All the proposed models exhibited high degree of prediction with regard to anti-tubercular activity. These models offer vast potential for providing lead structures for the development of potent therapeutic agents for treatment of tuberculosis.

## Figures and Tables

**Tab. 1.** Relationship between topological indices and antitubercular activity.

**Cpd. No.**	**Basic Ring**	**R**	*W_c_*	χ**^A^**	$\int_{c}^{P}$	$\xi_{c}^{P}$	**Antitubercular activity**				
							**Predicted Using MAA models**				**Reported**
							*W_c_*	χ**^A^**	$\int_{c}^{P}$	$\xi_{c}^{P}$	
1	A	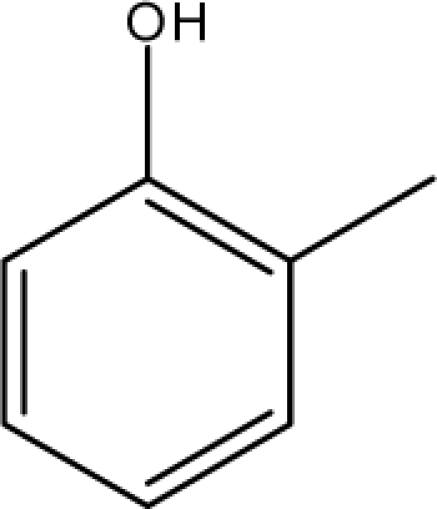	3896.417	13.461	7405.109	189780.156	±	+	–	±	+
2	A	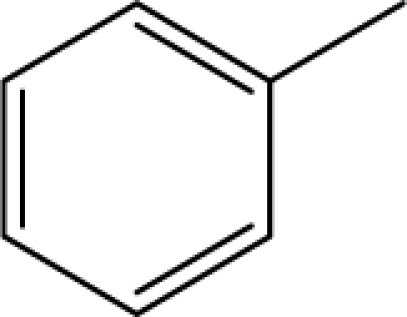	3583.748	13.117	2651.541	23721.924	–	–	–	–	–
3	A	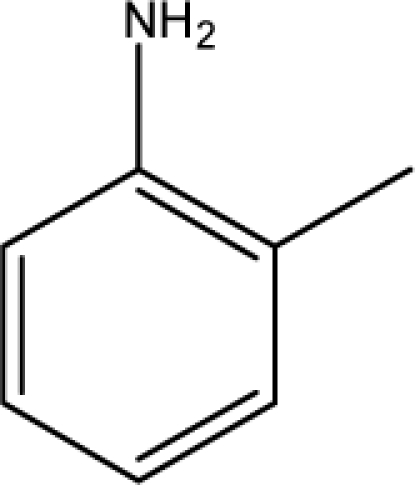	3893.845	13.493	7325.882	185842.313	±	+	–	±	–
4	A	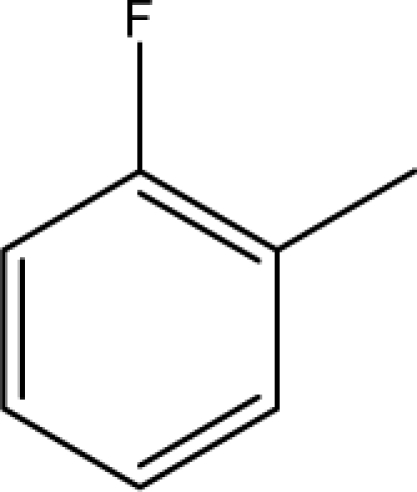	3900.292	13.416	7522.852	195710.641	±	+	–	±	+
5	A	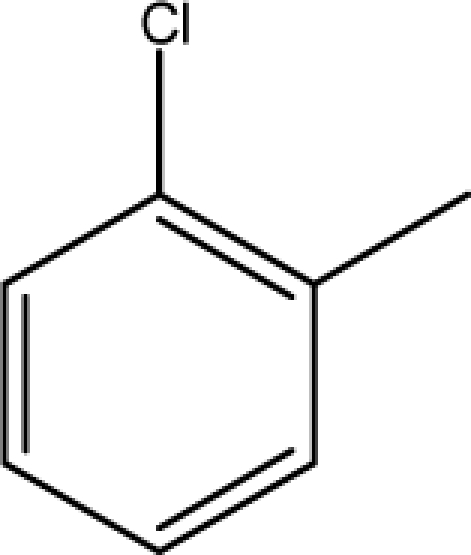	3738.752	13.471	2810.773	26456.617	–	–	–	–	–
6	A	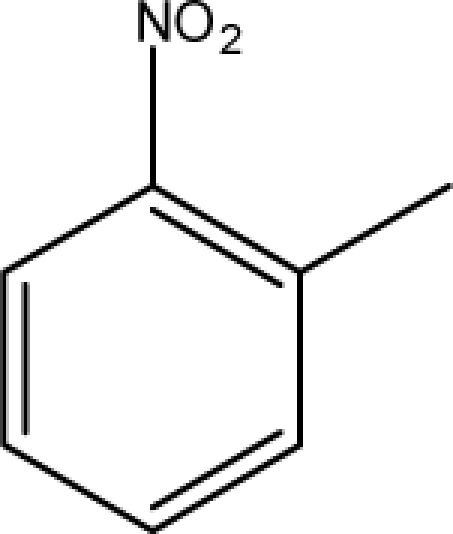	4596.538	14.191	29026.4	2752262.75	–	–	–	–	–
7	A	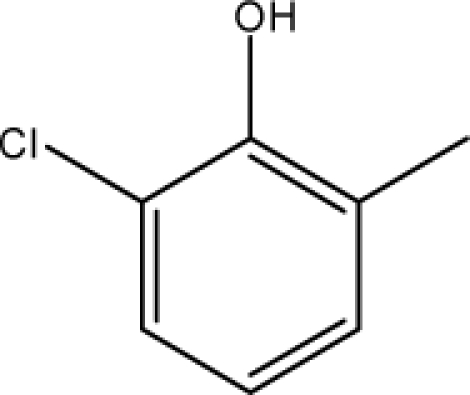	4263.42	13.586	7768.045	2337094.5	+	±	–	+	–
8	A	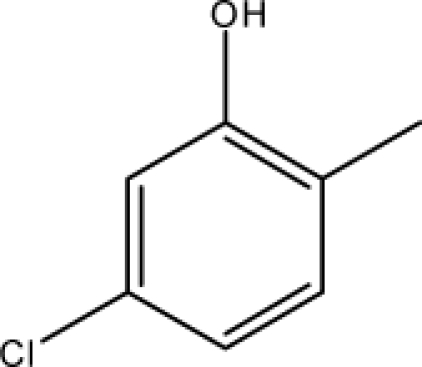	4289.42	13.545	8214.616	2233735	+	+	+	+	+
9	A	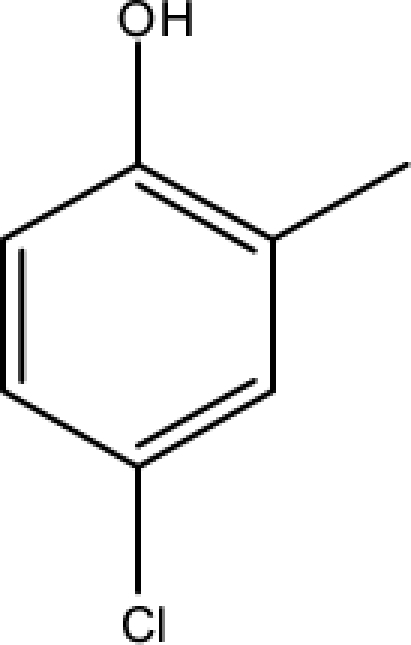	4265.42	13.545	7977.906	2335093	+	+	+	+	+
10	A	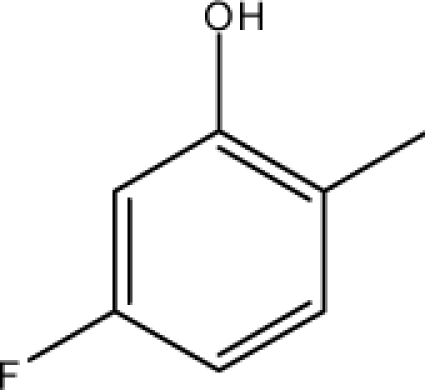	4267.42	13.736	28115.13	2313057.25	+	±	+	+	+
11	A	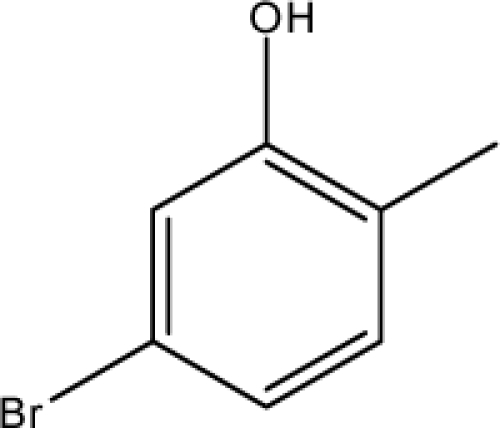	4248.865	13.45	8214.616	1821982.125	+	+	+	+	+
12	A	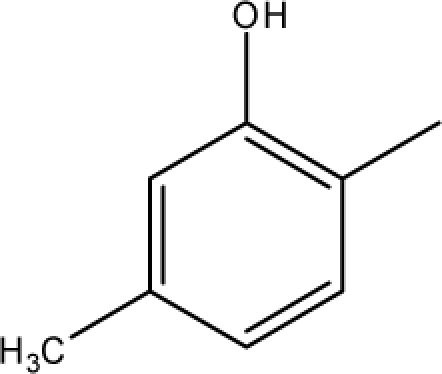	4258.092	13.854	27540.72	2353780.75	+	±	+	+	+
13	A	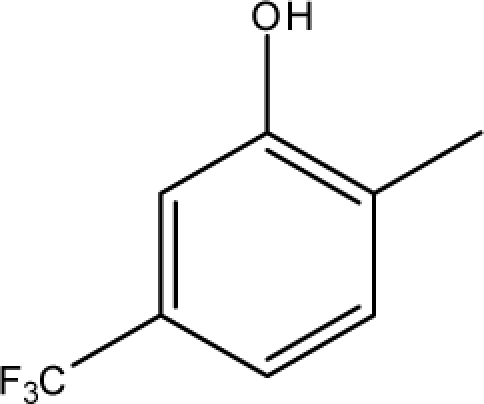	5478.722	14.769	554283.4	780373248	–	–	–	–	–
14	A	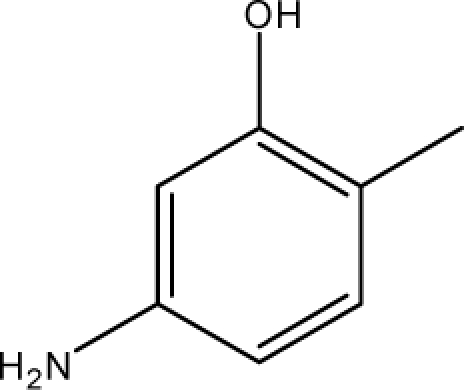	4260.764	13.817	27706.48	2341639	+	±	+	+	+
15	A	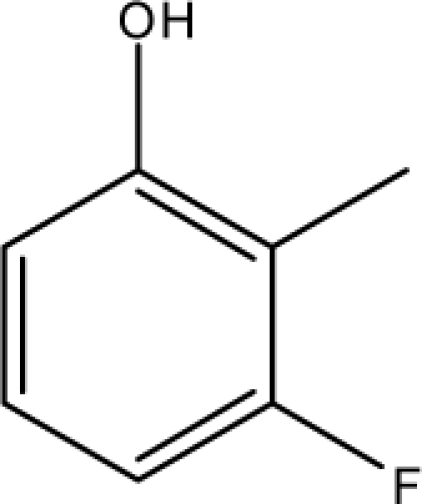	4217.42	13.759	26691.56	2453185.25	±	±	–	+	+
16	A	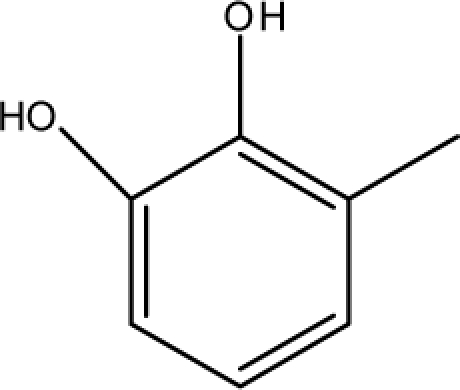	4237.42	13.808	27131.01	2452283.75	±	±	–	+	–
17	A	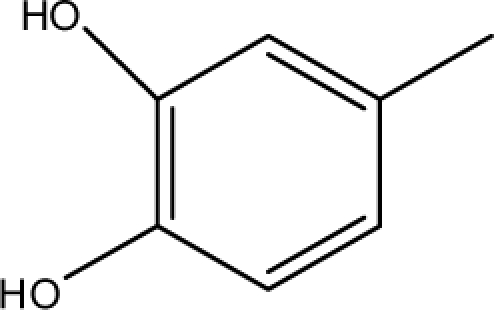	4287.42	13.788	28608.06	2462222.5	+	±	–	–	–
18	A	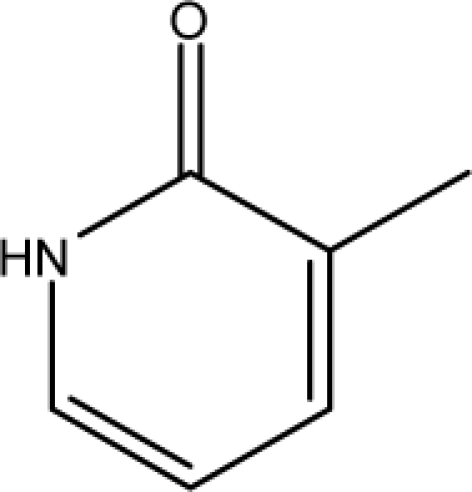	3899.34	13.391	7423.716	190558.016	±	–	–	±	–
19	A	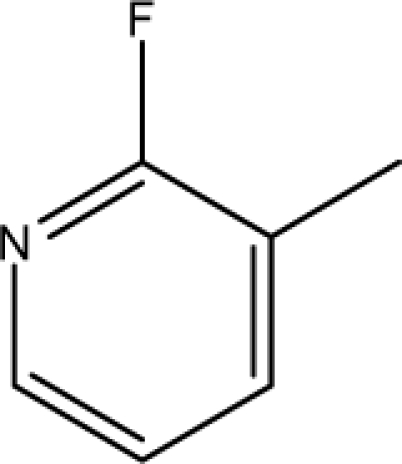	3903.215	13.35	7541.169	196488.5	±	–	–	±	–
20	A	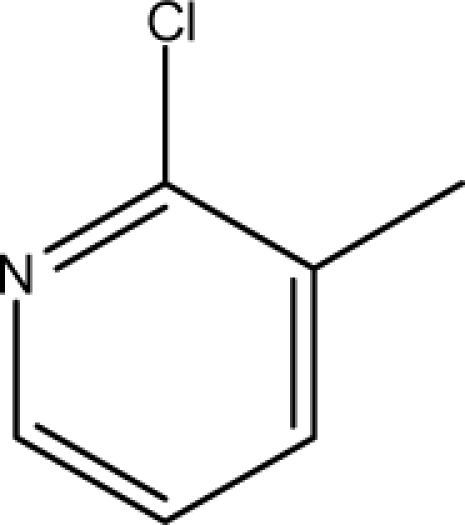	3924.528	13.179	2810.773	212818.438	±	–	–	±	–
21	A	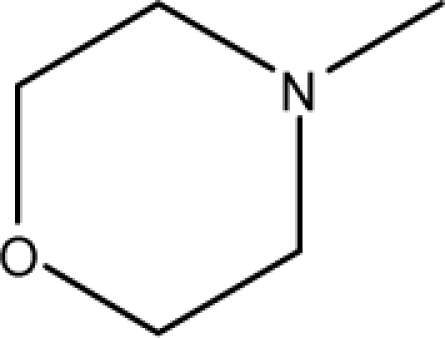	3612.958	12.875	2743.981	24494.674	–	–	–	–	–
22	A	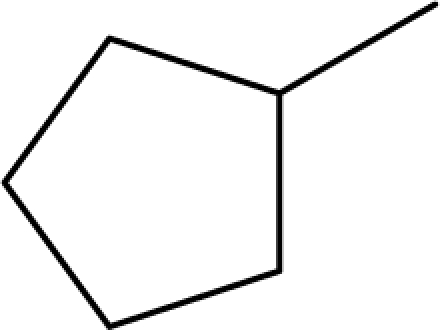	3254.241	12.617	2319.146	20258.762	–	–	–	–	–
23	A	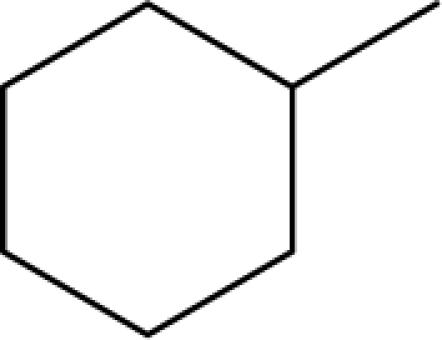	3583.748	13.117	2651.541	23721.924	–	–	–	–	–
24	A	H_3_C—	2119.209	10.527	5860.135	171240.594	–	–	–	–	–
25	A	H_3_C—O—	2400.371	10.93	6196.673	168013.875	–	–	–	–	–
26	A	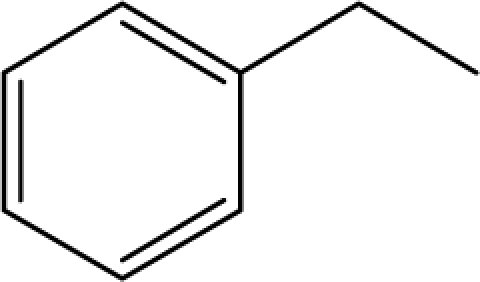	3986.914	13.588	3339.374	33262.125	±	±	–	–	–
27	A	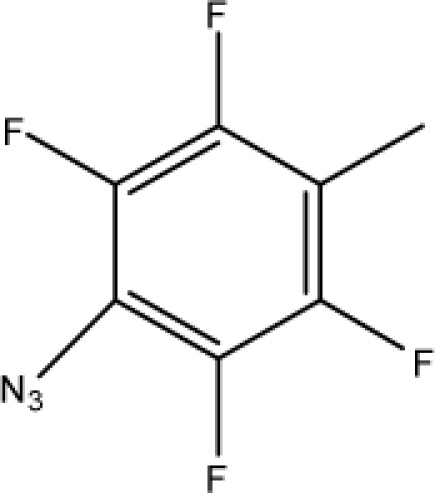	6181.416	15.608	2121377	10269211648	–	–	–	–	–
28	A	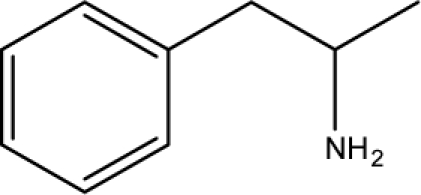	4731.027	14.477	10746.7	305689.281	–	–	–	±	–
29	B	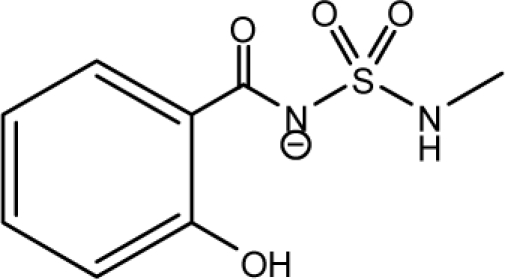	3855.002	13.503	7238.409	184644.953	±	±	–	±	+
30	B	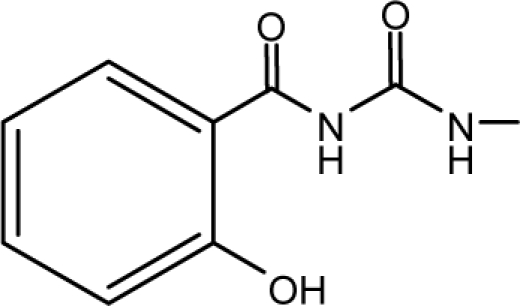	3210.494	13.974	1767.596	13858.688	–	–	–	–	–
31	B	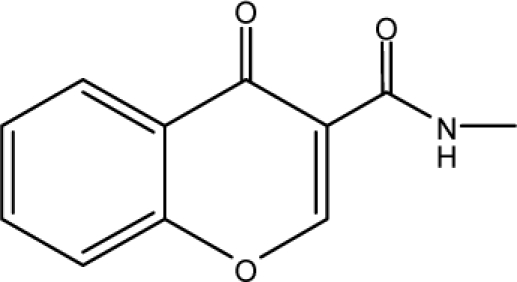	3331.263	14.574	676.29	2056.221	–	–	–	–	–

+…Active analogue; –…Inactive Analogue; ±…Transistional analogue where activity could not be specifically assigned; Note: The cation of anionic structures is Et_3_NH^+^ or Na^+^

**Tab. 2.** Topological indices.

**Code**	**Index**	**Reference**
A1	Molecular connectivity topochemical index	18, 19
A2	Eccentric adjacency topochemical index	37
A3	Augmented eccentric connectivity topochemical index	38
A4	Superadjacency topochemical index	39
A5	Eccentric connectivity topochemical index	23
A6	Connective eccentricity topochemical index	40
A7	Zagreb topochemical index, $M_{1}^{c}$	24
A8	Zagreb topochemical index, $M_{2}^{c}$	24
A9	Wiener’s topochemical index	41
A10	Superaugmented eccentric topochemical connectivity index1	42
A11	Superpendentic topochemical index	–
A12	Superaugmented eccentric topochemical connectivity index 3	42
A13	Pendentic eccentricity topochemical index	–
A14	Molecular connectivity index	13,43
A15	Eccentric adjacency index	44
A16	Augmented eccentric connectivity index	45
A17	Superadjacency index	39
A18	Eccentric connectivity index	20
A19	Connective eccentricity index	46
A20	Zagreb group parameter, M_1_	14, 15
A21	Zagreb group parameter, M_2_	14, 15
A22	Wiener’s index	47, 48
A23	Superaugmented eccentric connectivity index1	25
A24	Superpendentic index	49
A25	Eccentric distance sum index	50
A26	Pendentic eccentricity index	–

**Tab. 3.** Index values for all possible structures with three, four and five vertices containing one heteroatom and at least one pendent vertex.

**S.No.**	**Structure**	*W_c_*	χ**^A^**	$\int_{c}^{P}$	$\xi_{c}^{P}$
1	C—N—C	4.334	1.309	2.31	1.923
2	N—C—C	4.167	1.359	2.31	1.818
3	N—C—C—C	10.25	1.867	3.266	1.694
4	C—N—C—C	10.585	1.814	3.241	1.593
5	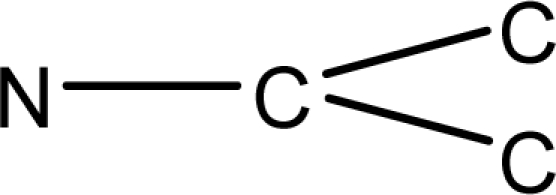	9.25	1.686	3.72	3.703
6	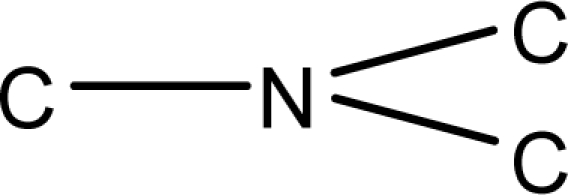	9.752	1.603	3.884	4
7	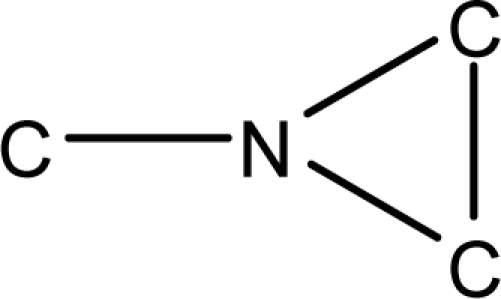	8.585	1.780	2.31	1.923
8	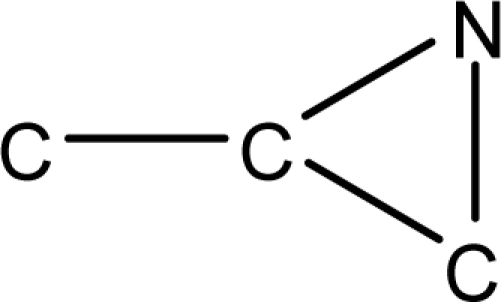	8.25	1.821	2.236	1.734
9	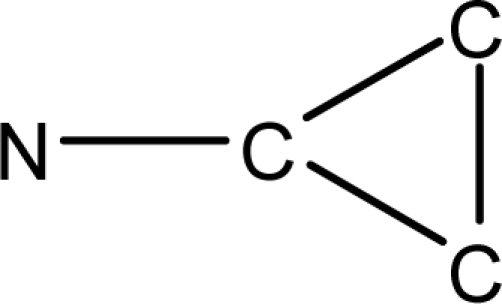	8.25	1.857	2.345	1.780
10	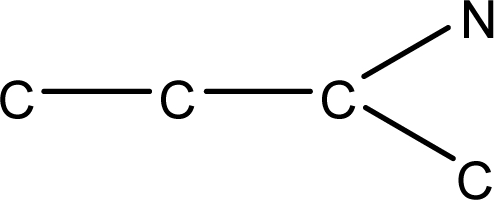	18.334	2.228	5.354	3.843
11	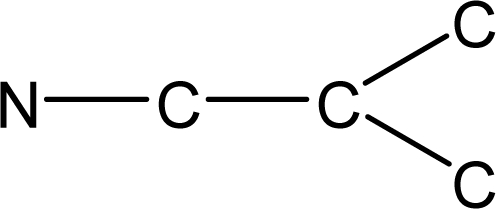	18.334	2.226	5.339	3.89
12	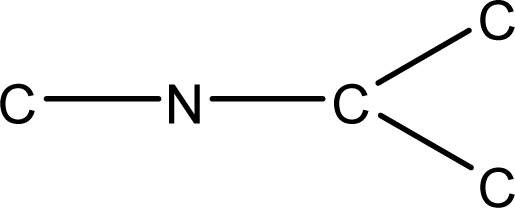	18.835	2.176	5.373	3.725
13	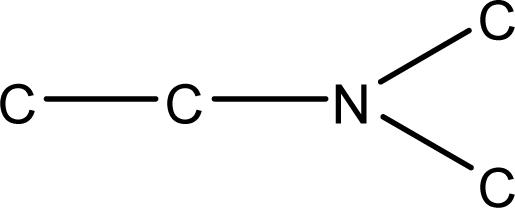	19.169	2.140	5.518	3.869
14	N—C—C—C—C	20.334	2.367	4.378	2.116
15	C—N—C—C—C	20.835	2.322	4.34	2.052
16	C—C—N—C—C	21.002	2.319	4.321	2.111
17	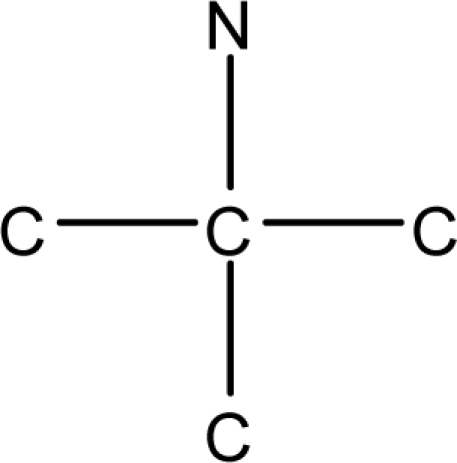	16.334	1.960	5.931	8.395
18	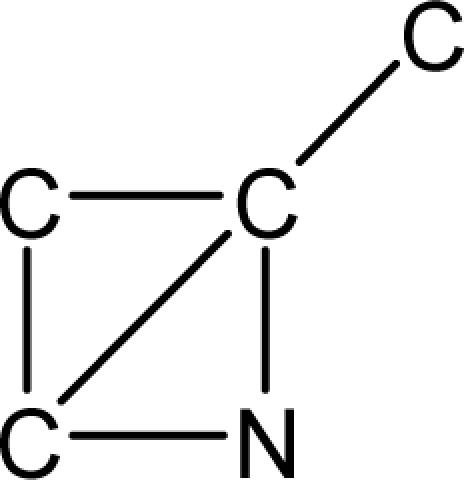	14.334	2.253	2.646	2.160
19	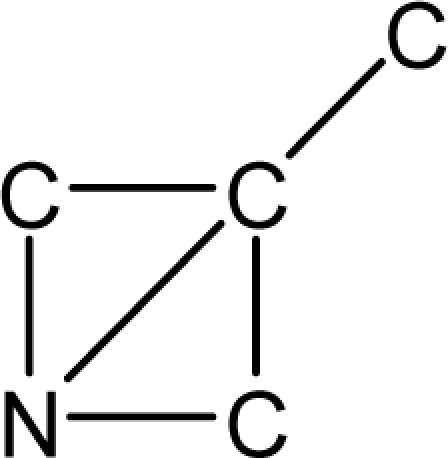	14.334	2.223	2.646	2.234
20	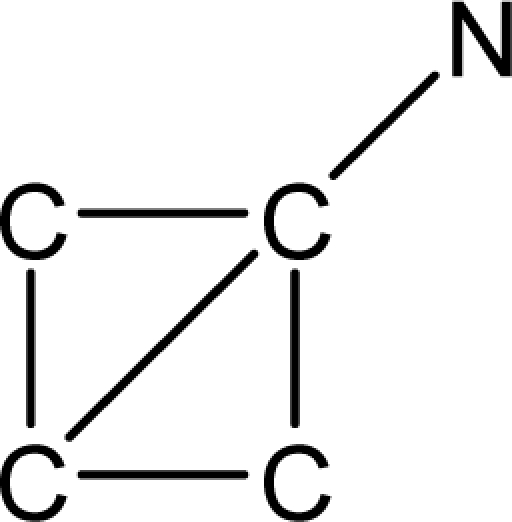	14.334	2.282	2.769	2.241
21	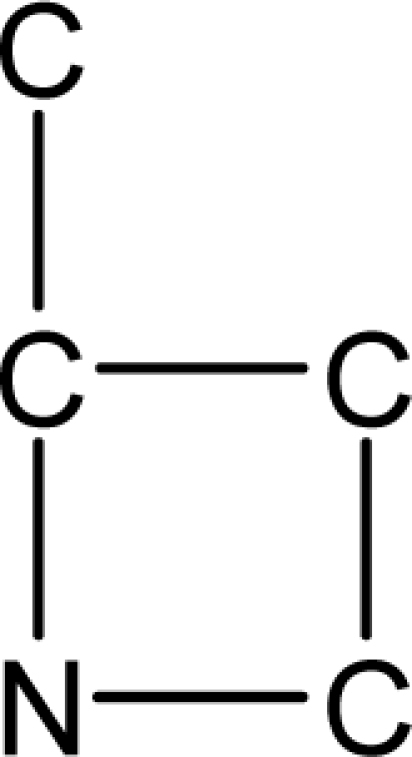	16.334	2.317	2.828	1.509
22	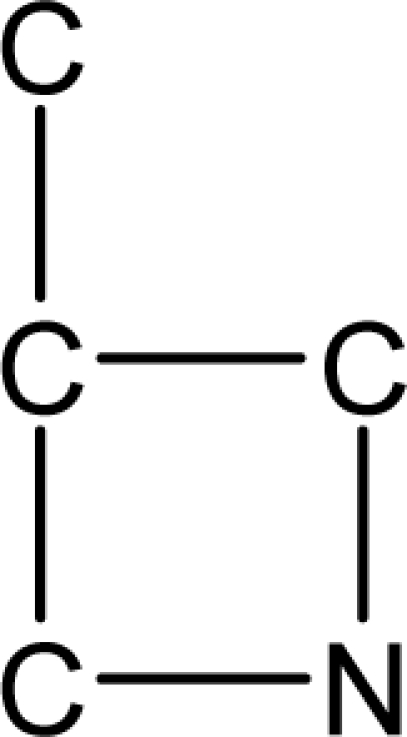	16.334	2.322	2.828	1.546
23	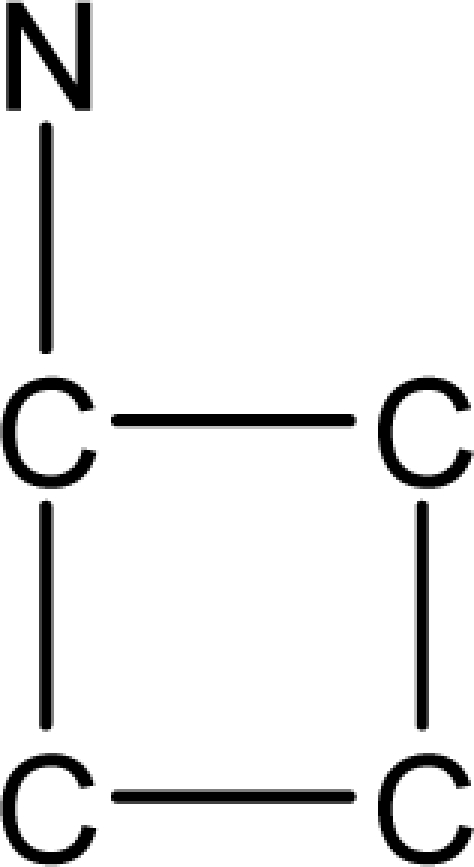	16.334	2.357	2.944	1.530
24	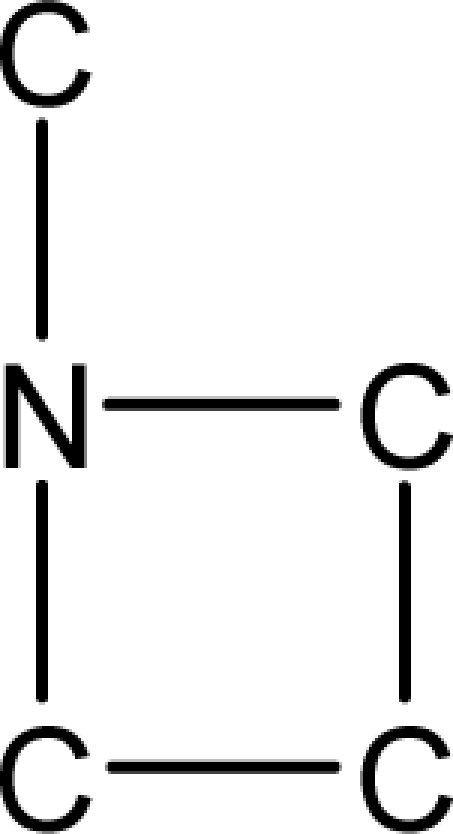	16.835	2.280	2.916	1.489
25	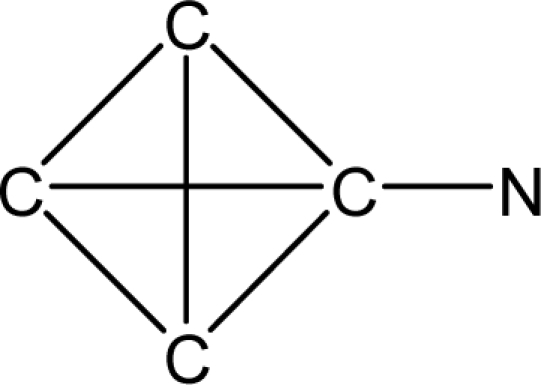	13.334	2.338	2.769	2.241
26	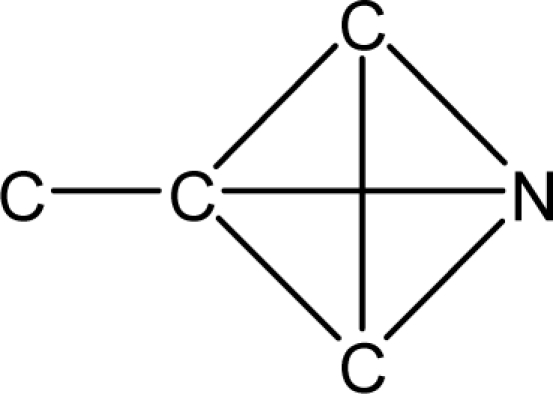	13.334	2.288	2.646	2.234
27	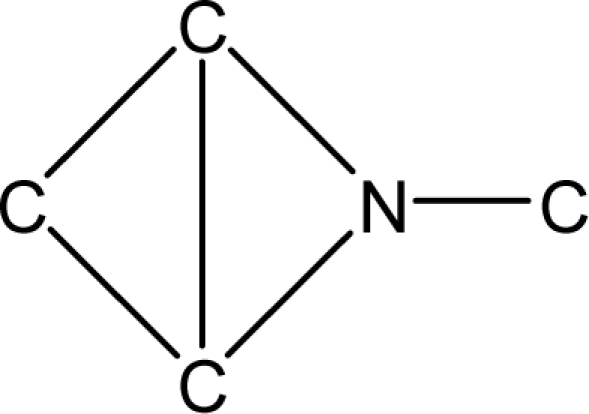	15.835	2.294	2.916	1.489
28	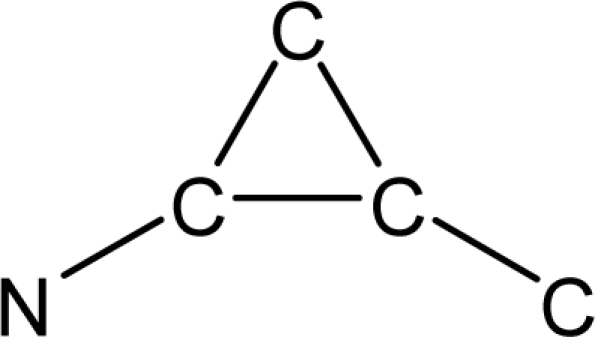	16.334	2.269	3.873	2.617
29	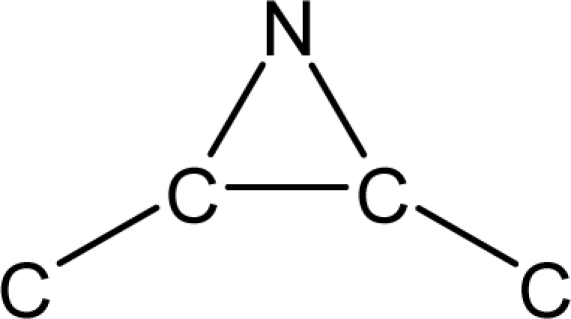	16.334	2.234	3.742	2.667
30	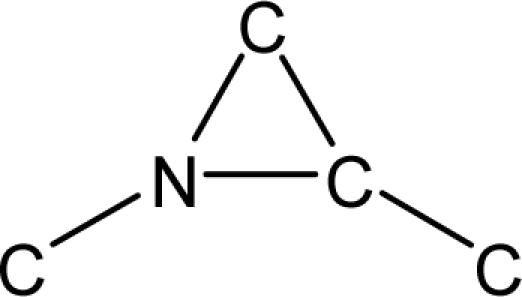	16.835	2.195	3.852	2.516
31	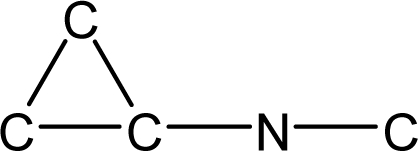	17.835	2.347	3.082	1.343
32	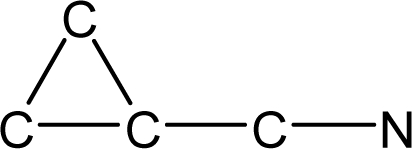	17.334	2.388	3.109	1.385
33	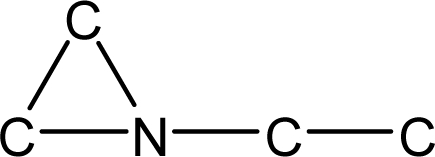	18.002	2.317	3.055	1.344
34	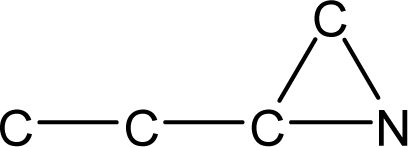	17.334	2.364	3	1.380
35	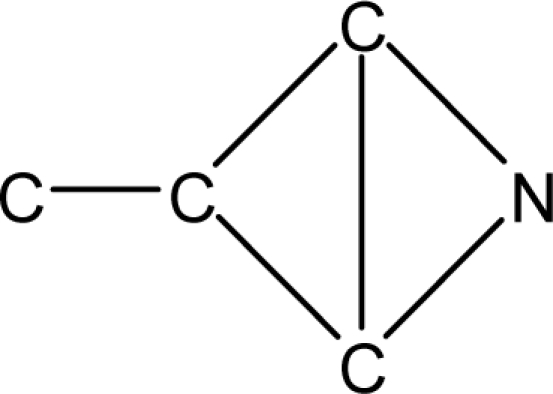	15.334	2.337	2.828	1.546
36	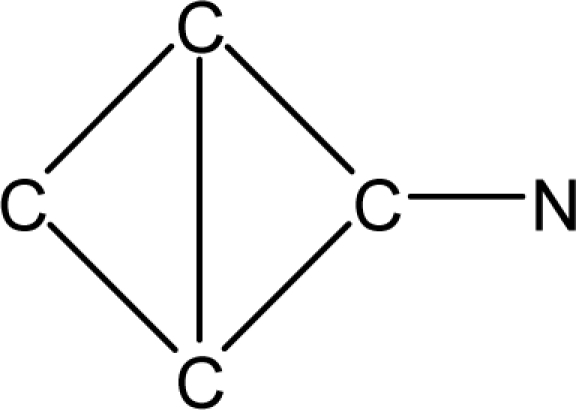	15.334	2.361	2.944	1.530
37	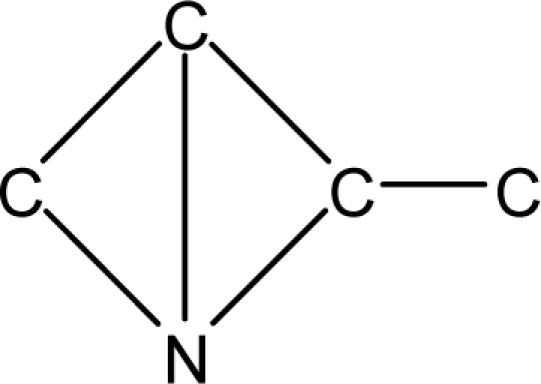	15.334	2.300	2.828	1.583
38	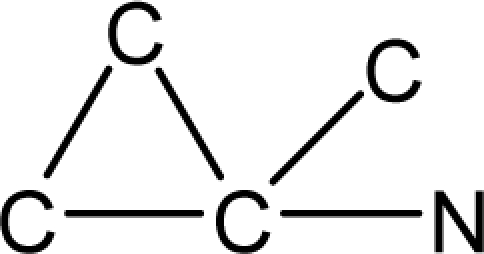	15.334	2.173	3.742	3.664
39	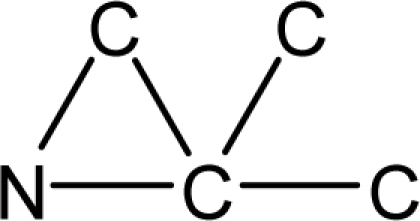	15.334	2.139	3.606	3.586

**Tab. 4.** Comparison of discriminating power and degeneracy of W*_c_*, χ^A^, $\int_{c}^{P}$, and $\xi_{c}^{P}$ using all possible structures having three, four and five vertices containing one heteroatom and one pendent vertex.

	*W_c_*	χ^A^	$\int_{c}^{P}$	$\xi_{c}^{P}$
*For three vertices*				
Minimum value	4.167	1.309	2.31	1.818
Maximum value	4.334	1.359	2.31	1.923
Ratio	1:1.04	1.1.04	1:1	1:1.05
Degeneracy	0/2	0/2	2/2	0/2
*For four vertices*				
Minimum value	8.25	1.603	2.236	1.593
Maximum value	10.585	1.867	3.884	4
Ratio	1:1.28	1.16	1:1.73	1:2.51
Degeneracy	1/7	0/7	0/7	0/7
*For five vertices*				
Minimum value	13.334	1.96	2.646	1.343
Maximum value	21.002	2.388	5.931	8.395
Ratio	1:1.57	1:1.22	1:2.24	1:6.25
Degeneracy	13/30	2/30	9/30	5/30

Degeneracy = Number of compounds having same values / total number of compounds with same number of vertices.

**Tab. 5.** Confusion Matrix for antitubercular activity and recognition rate of models based on decision tree and Random forest.

**Model**	**Description**	**Ranges**	**Number of compounds Predicted**		**Sensitivity (%)**	**Specificity (%)**	**Overall Accuracy of Prediction**	**MCC**
			Active	Inactive				
Decision Tree	Training set	Active	10	0	100	100	100	1
		Inactive	0	21				
	Cross validated set	Active	07	03	70	80.9	77.4	0.497
		Inactive	04	17				

Random Forest		Active	5	5	50	76.19	67.74	0.26
		Inactive	16	5				

**Tab. 6.** Intercorrelation matrix.

	*W_c_*	χ**^A^**	$\int_{c}^{P}$	$\xi_{c}^{P}$	${\text{M}}_{1}^{\text{c}}$	${\text{M}}_{2}^{\text{c}}$	^A^*ξ^c^*
*W_c_*	1	0.851	0.63	0.57	0.914	0.847	0.336
χ^A^		1	0.476	0.435	0.617	0.544	−0.074
$\int_{c}^{P}$			1	0.984	0.594	0.417	0.282
$\xi_{c}^{P}$				1	0.522	0.363	0.271
$M_{1}^{c}$					1	0.923	0.632
$M_{2}^{c}$						1	0.653
^A^*ξ^c^*							1

**Tab. 7.** MAA derived topological models for antitubercular activity.

**Model Index**	**Nature of range in proposed model**	**Index value**	**Number of analogues falling in the range**		**Percent accuracy**	**Average MIC_99_ (μM) (Correctly predicted analogues)**	**Overall accuracy of prediction (%)**
			**Total**	**Correct**			
*W_c_*	Lower Inactive	<3855.002	9	9	>99.9	43.22	90.4
	Transitional	3855.002–<4248.865	10	N.A.	N.A.	15.61	
	Active	4248.865–4289.42	8	6	75	0.019	
	Upper Inactive	>4289.42	4	4	>99.9	37.05	

χ^A^	Lower Inactive	<13.416	9	9	>99.9	30.71	91.3
	Active	13.416–13.545	8	6	75	0.016	
	Transitional	>13.545–≤13.854	8	N.A.	N.A.	20.1	
	Upper Inactive	>13.854	6	6	>99.9	41.5	

$\int_{c}^{P}$	Lower Inactive	< 7238.409	11	11	>99.9	44.47	91.6
	Transitional	7238.409–<7977.906	7	N.A.	N.A.	1.07	
	Active	7977.906–28115.13	9	7	77.7	0.018	
	Upper Inactive	> 28115.13	4	4	>99.9	52.93	

$\xi_{c}^{P}$	Lower Inactive	<184644.953	10	10	>99.9	48.9	91.3
	Transitional	184644.953–<1821982.125	8	N.A.	N.A.	5.51	
	Active	1821982.125–2453185.25	9	7	>77.7	0.018	
	Upper Inactive	>2453185.25	4	4	>99.9	52.92	

**Tab. 8.** MAA derived topological models for antitubercular activity in Iron-deficient state.

**Model Index**	**Nature of range in proposed model**	**Index value**	**Number of analogues falling in the range**		**Percent accuracy**	**Average MIC_99_ (μM) (Correctly predicted analogues)**	**Overall accuracy of prediction (%)**
			**Total**	**Correct**			
*W_c_*	Lower Inactive	<3855.002	9	7	>77.7	200	80.9
	Transitional	3855.002–<4248.865	10	N.A.	N.A.	103.19	
	Active	4248.865–4289.42	8	6	75	5.74	
	Upper Inactive	>4289.42	4	4	>99.9	200	

χ^A^	Lower Inactive	<13.416	9	7	>77.7	200	78.0
	Active	13.416–13.545	8	6	75	7.38	
	Transitional	>13.545–≤13.854	8	N.A.	N.A.	86.02	
	Upper Inactive	>13.854	6	5	83	200	

$\int_{c}^{P}$	Lower Inactive	< 7238.409	11	8	72.72	200	79.16
	Transitional	7238.409 – <7977.906	7	N.A.	N.A.	94.73	
	Active	7977.906 – 28115.13	9	7	77.7	5.03	
	Upper Inactive	> 28115.13	4	4	>99.9	200	

$\xi_{c}^{P}$	Lower Inactive	<184644.953	10	8	80	200	82.6
	Transitional	184644.953–<1821982.125	8	N.A.	N.A.	103.19	
	Active	1821982.125–2453185.25	9	7	77.7	5.03	
	Upper Inactive	>2453185.25	4	4	>99.9	200	

**Tab. 9.** MAA derived topological models for antitubercular activity in Iron-rich state.

**Model Index**	**Nature of range in proposed model**	**Index value**	**Number of analogues falling in the range**		**Percent accuracy**	**Average MIC_99_ (μM) (Correctly predicted analogues)**	**Overall accuracy of prediction (%)**
			**Total**	**Correct**			
*W_c_*	Lower Inactive	<3855.002	9	7	>77.7	200	80.9
	Transitional	3855.002–<4248.865	10	N.A.	N.A.	112.74	
	Active	4248.865–4289.42	8	6	75	33.39	
	Upper Inactive	>4289.42	4	4	>99.9	200	

χ^A^	Lower Inactive	<13.416	9	7	77.7	200	78
	Active	13.416–13.545	8	6	75	33.65	
	Transitional	>13.545–≤13.854	8	N.A.	N.A.	110.35	
	Upper Inactive	>13.854	6	5	83	200	

$\int_{c}^{P}$	Lower Inactive	< 7238.409	11	8	72.72	200	79.16
	Transitional	7238.409–<7977.906	7	N.A.	N.A.	121.7	
	Active	7977.906–28115.13	9	7	77.7	29.07	
	Upper Inactive	> 28115.13	4	4	>99.99	200	

$\xi_{c}^{P}$	Lower Inactive	<184644.953	10	8	80	200	82.6
	Transitional	184644.953–<1821982.125	8	N.A.	N.A.	112.74	
	Active	1821982.125–2453185.25	9	7	77.7	29.07	
	Upper Inactive	>2453185.25	4	4	>99.9	200	
